# Inference of COVID-19 epidemiological distributions from Brazilian hospital data

**DOI:** 10.1098/rsif.2020.0596

**Published:** 2020-11-25

**Authors:** Iwona Hawryluk, Thomas A. Mellan, Henrique Hoeltgebaum, Swapnil Mishra, Ricardo P. Schnekenberg, Charles Whittaker, Harrison Zhu, Axel Gandy, Christl A. Donnelly, Seth Flaxman, Samir Bhatt

**Affiliations:** 1 MRC Centre for Global Infectious Disease Analysis, Abdul Latif Jameel Institute for Disease and Emergency Analytics (J-IDEA), School of Public Health, Imperial College London, London, UK,; 2 Department of Mathematics, Imperial College London, London SW7 2AZ, UK; 3 Nuffield Department of Clinical Neurosciences, Oxford, UK; 4 Department of Statistics, University of Oxford, Oxford, UK

**Keywords:** COVID-19, Brazil, symptom-onset-to-death, admission-to-death, model selection

## Abstract

Knowing COVID-19 epidemiological distributions, such as the time from patient admission to death, is directly relevant to effective primary and secondary care planning, and moreover, the mathematical modelling of the pandemic generally. We determine epidemiological distributions for patients hospitalized with COVID-19 using a large dataset (*N* = 21 000 − 157 000) from the Brazilian Sistema de Informação de Vigilância Epidemiológica da Gripe database. A joint Bayesian subnational model with partial pooling is used to simultaneously describe the 26 states and one federal district of Brazil, and shows significant variation in the mean of the symptom-onset-to-death time, with ranges between 11.2 and 17.8 days across the different states, and a mean of 15.2 days for Brazil. We find strong evidence in favour of specific probability density function choices: for example, the gamma distribution gives the best fit for onset-to-death and the generalized lognormal for onset-to-hospital-admission. Our results show that epidemiological distributions have considerable geographical variation, and provide the first estimates of these distributions in a low and middle-income setting. At the subnational level, variation in COVID-19 outcome timings are found to be correlated with poverty, deprivation and segregation levels, and weaker correlation is observed for mean age, wealth and urbanicity.

## 1. Introduction

Surveillance of COVID-19 has progressed from initial reports on 31 December 2019 of pneumonia with unknown aetiology in Wuhan, China [1], to the confirmation of 9826 cases of SARS-CoV-2 across 20 countries one month later [2], to the current pandemic of greater than 28 million confirmed cases and 900 000 deaths globally to date at the time of writing [3]. Early estimates of epidemiological distributions provided critical input that enabled modelling to identify the severity and infectiousness of the disease. The onset-to-death distribution [4,5], characterizing the range of times observed between the onset of first symptoms in a patient and their death, proved crucial in early estimates of the infection fatality ratio (IFR) where it was used to estimate the cumulative number of deaths in the beginning of the epidemic in Wuhan [6]. Similarly, the onset-to-death distribution was used in recent approaches to modelling the transmission dynamics of SARS-CoV-2 to estimate the reproduction number *R*_*t*_ and other important epidemiological quantities such as the serial interval distribution [7–12].

Initial estimates of COVID-19 epidemiological distributions necessarily relied on relatively few data points, with the events comprising these distributions occurring over a period of time that was short compared to the temporal pathologies of the disease progression, resulting in wide confidence or credible intervals and a sensitivity to time-series censoring effects [6]. Global surveillance of the disease over the past 197 days has provided more data to re-evaluate the time-delay distributions of the disease. In particular, public availability of a large number of patient-level hospital records—over 390 000 in total at the time of writing—from the SIVEP-Gripe (*Sistema de Informação de Vigilância Epidemiológica da Gripe*) database published by Brazil’s Ministry of Health [13], provides an opportunity to make robust statistical estimates of the onset-to-death and other time-delay distributions such as onset-to-diagnosis, length of ICU stay, onset-to-hospital-admission, onset-to-hospital-discharge, onset-to-ICU-admission and hospital-admission-to-death. In this work, we fit and present an analysis of these epidemiological distributions, with the paper set out as follows. Section 2 describes the data used from the SIVEP-Gripe database [13], and the methodological approach applied to fit the distributions using a hierarchical Bayesian model with partial pooling. Section 3 provides a description of the results from this study from fitting epidemiological distributions at national and subnational level to a range of probability density functions (PDFs). The results are discussed in §4, including associations with socio-economic factors, such as education, segregation and poverty, and conclusions are given in §5.

## 2. Methods

### 2.1. Data

The SIVEP-Gripe database provides detailed patient-level records for all individuals hospitalized with severe acute respiratory illness, including all suspected or confirmed cases of severe COVID-19 reported by both private and public sector healthcare institutions, from small rural hospitals to large metropolitan academic centres [13–17]. The records include the date of admission, date of onset of symptoms, state where the patient lives, state where they are being treated, and date of outcome (death or discharge), among other diagnosis related variables. We extracted the data for confirmed COVID-19 records starting on 25 February 2020 and considered records in our analysis ending on 7 July 2020. The dataset was filtered to obtain rows for onset-to-death, hospital-admission-to-death, length of ICU stay, onset-to-hospital-admission, onset-to-hospital-discharge, onset-to-ICU-admission and onset-to-diagnosis. Onset-to-diagnosis data were split into the diagnosis confirmed by PCR and those confirmed by other methods, such as rapid antibody and antigen tests, called non-PCR throughout this manuscript. Entries resulting in distribution times greater than 133 days were considered a typing error and removed, as the first recorded COVID-19 case in Brazil was on 25 February [18].

Additional filtering of the data was applied for onset-to-ICU-admission, onset-to-hospital-admission and onset-to-death in order to eliminate bias introduced by potentially erroneous entries identified in the data for these distributions. We removed the rows where admission to the hospital or ICU or death happened on the same day as onset of symptoms, assuming that these were actually incorrectly inputted entries. The decision to test removing the first day is motivated firstly by the observation of a number of conspicuous data entry errors in the database, and secondly by anomalous spikes corresponding to same-day events observed in these distributions. An example of the anomalous spikes in the onset-to-death distribution is shown in appendix B, figure 5 for selected states.

Sensitivity analyses on data inclusion, regarding the removal of anomalous spikes in first-day data indicative of reporting errors (e.g. in onset-to-hospital-admission), and regarding the sensitivity of the dataset to time-series censoring effects, are set out in the results §3.3.

**Figure 1.**
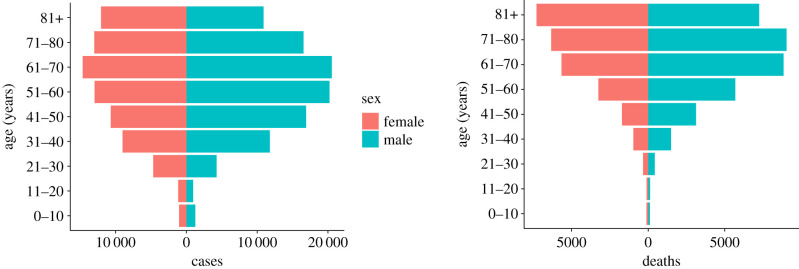
Demography of COVID-19 patients in Brazil. The left plot shows the number of confirmed COVID-19 cases and the right shows the number of confirmed COVID-19 deaths. Data were extracted from the SIVEP-Gripe database from 25 February 2020 up to 7 July 2020 [13].

A summary of the data, including number and a range of samples per variable from the SIVEP-Gripe dataset is given in table 1. The age-sex structure of hospitalized patients in the database with confirmed COVID-19 diagnoses is presented in figure 1. A breakdown of the number of data samples per state is provided in appendix B, table 5.

**Table 1.** Summary of the distribution data extracted from SIVEP-Gripe database [13]. Number of samples (*N*_samples_) is given for the whole country.

distribution	*N*_samples_	range (days)
onset-to-death	59 271	1–114
hospital-admission-to-death	52 821	0–99
ICU-stay	21 709	0–89
onset-to-hospital-admission	141 618	1–129
onset-to-hospital-discharge	69 478	0–120
onset-to-ICU-admission	46 617	0–101
onset-to-diagnosis (PCR)	156 558	0–129
onset-to-diagnosis (non-PCR)	19 438	0–102

### 2.2. Model fitting

Gamma, Weibull, lognormal, generalized lognormal [19] and generalized gamma [20] PDFs are fitted to several epidemiological distributions, with the specific parameterizations provided in appendix B, table 4. The parameters of each distribution are fitted in a joint Bayesian hierarchical model with partial pooling, using data from the 26 states and one federal district of Brazil, extracted and filtered to identify specific epidemiological distributions such as onset-to-death, ICU-stay, and so on.

As an example consider fitting a gamma PDF for the onset-to-death distribution. The gamma distribution for the *i*^th^ state is given by

2.1 $$\text{Gamma}(\alpha_{i},\beta_{i}),$$

where shape and scale parameters are assumed to be positively constrained, normally distributed random variables

2.2 $$\alpha_{i}∼N(\alpha_{\mathrm{Brazil}},\sigma_{1})$$

and

2.3 $$\beta_{i}∼N(\beta_{\mathrm{Brazil}},\sigma_{2}).$$

The parameters *α*_Brazil_ and *β*_Brazil_ denote the national level estimates, and

2.4 $$\sigma_{1}∼N^{+}(0,1)\;\text{and}\;\sigma_{2}∼N^{+}(0,1),$$

where *N*^+^( · ) is a truncated normal distribution. In this case, parameters *α*_Brazil_ and *β*_Brazil_ are estimated by fitting a gamma PDF to the fully pooled data, that is including the observations for all states. Prior probabilities for the national level parameters for each of the considered PDFs are chosen to be *N*^+^(0, 1). The only exception was for the more complex generalized gamma distribution which used more informed priors to speed-up fitting. The priors for the generalized gamma distribution were chosen based on the previous fits to be: *μ*_Brazil_ ∼ *N*^+^(2, 0.5), *σ*_Brazil_ ∼ *N*^+^(0.5, 0.5) and *s*_Brazil_ ∼ *N*^+^ (1.5, 0.5). Additionally, for all fitted densities, the mean and variance parameters were constrained to be positive.

Posterior samples of the parameters in the model are generated using Hamiltonian Monte Carlo (HMC) with Stan [21,22]. For each fit, we use four chains and 2000 iterations, with half of the iterations dedicated to warm-up.

The preference for one fitted model over another is characterized in terms of the Bayesian support, with the model evidence calculated to see how well a given model fits the data, and comparison between two models using Bayes Factors (BFs). BFs provide a principled fully Bayesian approach to select between models, incorporating the full posterior densities and thus also the uncertainty of each of the parameters instead of point estimates [23–26]. Moreover, BFs naturally balance the complexity and accuracy of the compared models, ensuring that the excessively complex models are not automatically favoured. Historically simpler methods have been favoured, as BFs can be costly to compute for complex models, however using recent efficient methods this is not an issue [27]. The details of how to estimate the model evidence and calculate the BF for each pair of models are given in appendix A.

Data cleaning and the analysis of the results was conducted using Python (v. 3.7.7) programming language [28]. The PyStan (v. 2.19.0.0) interface was used for running model fitting with Stan [29].

## 3. Results

### 3.1. Brazilian epidemiological distributions

**Figure 2.**
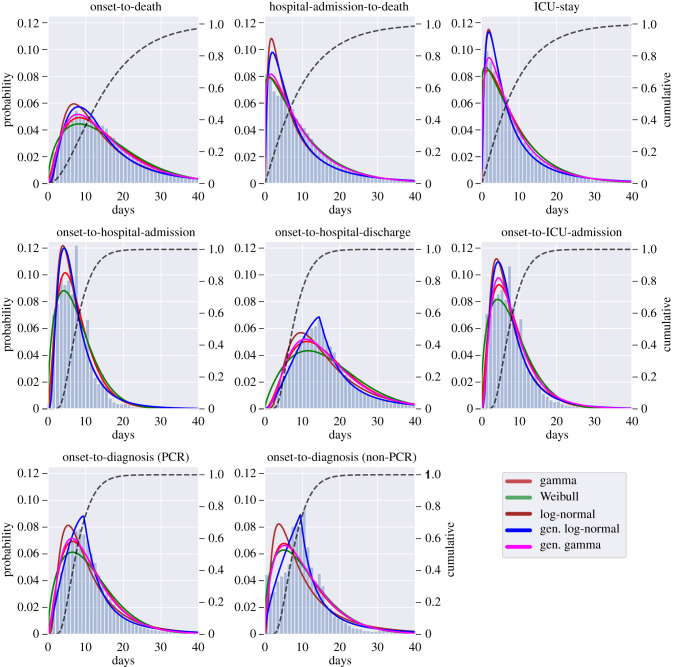
Histograms for onset-to-death, hospital-admission-to-death, ICU-stay, onset-to-hospital-admission, onset-to-hospital-discharge, onset-to-ICU-admission onset-to-diagnosis (PCR) and onset-to-diagnosis (non-PCR) distributions show data for Brazil extracted from the SIVEP-Gripe database [13]. For each distribution, solid lines are for fitted PDFs and the dashed line shows the cumulative distribution function of the best-fitting PDF. The left-hand side *y*-axis gives the probability value for the PDFs and the right-hand side *y*-axis shows the value for the cumulative distribution function. All values on the *x*-axes are given in days. State-level fits are shown in figure 3 and appendix B, figures 6 and 7.

Five trial PDFs—gamma, Weibull, lognormal, generalized lognormal and generalized gamma—were fitted to the epidemiological data shown in figure 2.

**Figure 3.**
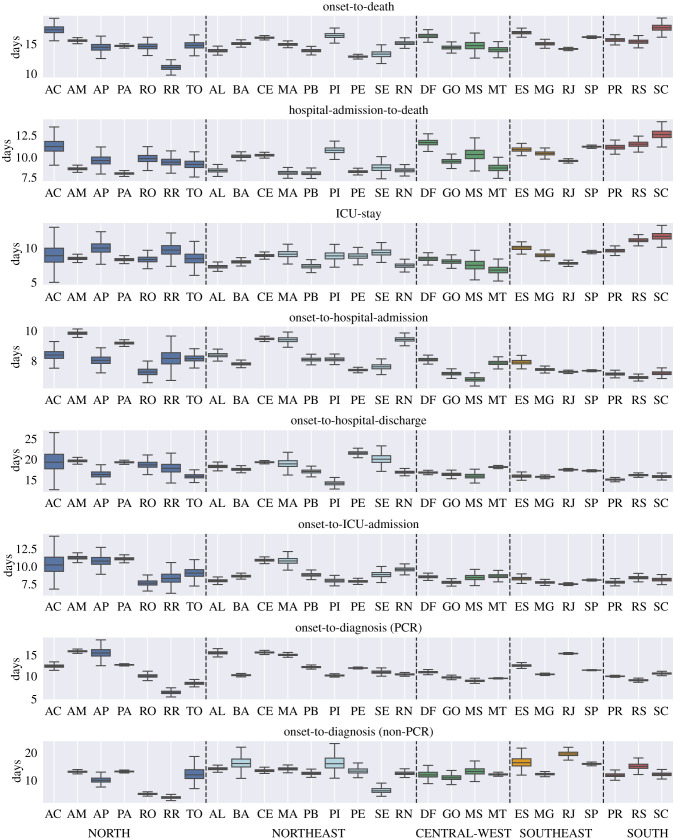
Estimates of the mean time in days for onset-to-death, hospital-admission-to-death and each of the other distributions fitted in the joint model of Brazil. Estimates are grouped by the five regions of Brazil, North (blue), Northeast (light-blue), Central-West (green), Southeast (orange), South (red), and are shown for Acre (AC), Amazonas (AM), Amapá (AP), Pará (PA), Rondônia (RO), Roraima (RR), Tocantins (TO), Alagoas (AL), Bahia (BA), Ceará (CE), Maranhão (MA), Paraíba (PB), Piauí (PI), Pernambuco (PE), Sergipe (SE), Rio Grande do Norte (RN), Distrito Federal (DF), Goiás (GO), Mato Grosso do Sul (MS), Mato Grosso (MT), Espírito Santo (ES), Minas Gerais (MG), Rio de Janeiro (RJ), São Paulo (SP), Paraná (PR), Rio Grande do Sul (RS), Santa Catarina (SC). For state Acre, the onset-to-diagnosis (non-PCR) mean diverged due to the small number of samples (*n*=1). The full posterior distribution for each mean estimate is given in appendix B, figures 6 and 7.

**Figure 4.**
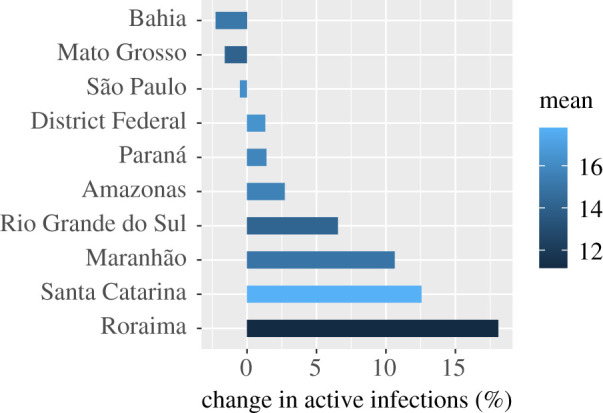
This figure shows the percentage change in active infections, estimated on 23 June 2020 using the COVID-19 model of Flaxman *et al.* [7] that results from using state-specific onset-to-death distributions (see appendix B, table 7) compared to a single national-level one. The effect for each state is coloured according to the mean of the state’s onset-to-death gamma distribution, given in days. The mean onset-to-death for Brazil is 15.2 days.

**Figure 5.**
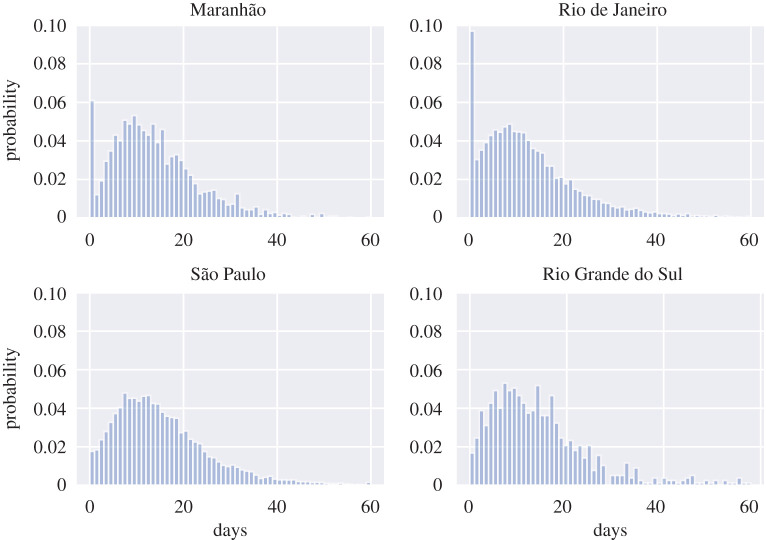
Distribution of onset-to-death for Maranhão, Rio de Janeiro, São Paulo and Rio Grande do Sul. Anomalous spikes for the first day can be observed for Maranhão and Rio de Janeiro, indicating they might be a reporting error.

**Figure 6.**
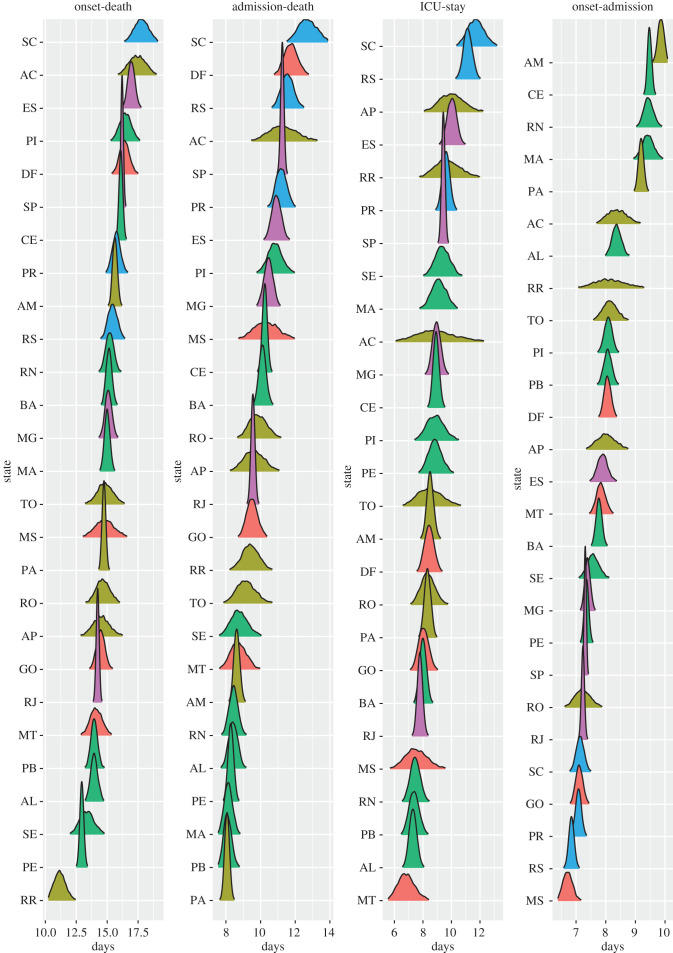
Posterior distribution of mean times (in days) for onset-to-death, hospital-admission-to-death, ICU stay and onset-to-hospital-admission, sorted by mean value. Plots are colour-coded by the geographical region which the state belongs to: North (yellow), Northeast (green), Central-West (orange), Southeast (purple) and South (blue).

**Figure 7.**
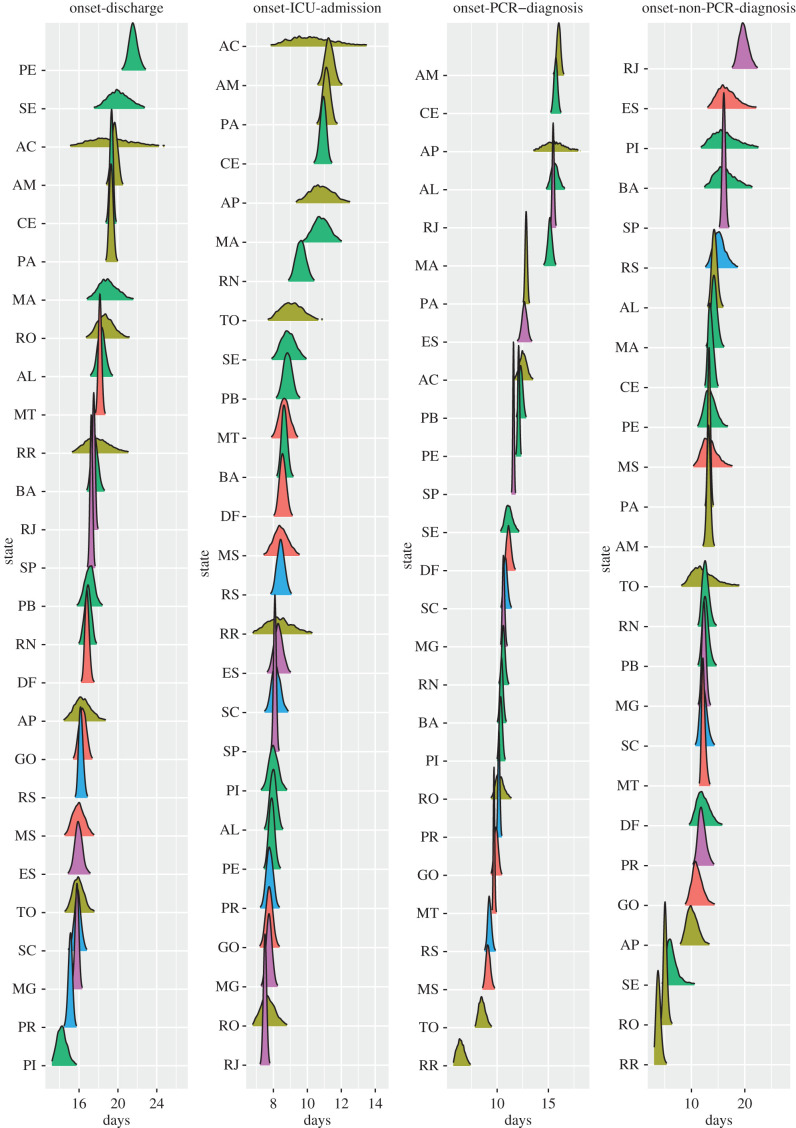
Posterior distribution of mean times (in days) for onset-to-hospital-discharge, onset-to-ICU-admission, onset-to-diagnosis (PCR) and onset-to diagnosis (non-PCR), sorted by mean value. Plots are colour-coded by the geographical region which the state belongs to: North (yellow), Northeast (green), Central-West (orange), Southeast (purple) and South (blue).

All of the models’ fits were tested by using the BFs based on the Laplace approximation and corrected using thermodynamic integration [27,30,31], as described in appendix A. The thermodynamic integration contribution was negligible suggesting the posterior distributions are satisfactorily approximated as multivariate normal. The conclusions on the preferred PDF were not sensitive to the choice of prior distributions, that is the preferred model was still the favoured one even when more informative prior distributions were applied for all PDFs. The BFs used for model selection are shown in appendix B, table 6.

The gamma PDF provided the best fit to the onset-to-death, hospital-admission-to-death and ICU-stay data. For the remaining distributions—onset-to-diagnosis (non-PCR), onset-to-diagnosis (PCR), onset-to-hospital-discharge, onset-to-hospital-admission and onset-to-ICU-admission—the generalized lognormal distribution was the preferred model. The list of preferred PDFs for each distribution, together with the estimated mean, variance and PDFs’ parameter values for the national fits are given in table 2. The 95% credible intervals (CrI) for parameters of each of the preferred PDFs was less than 0.1 wide, therefore in table 2 we show only point estimates.

**Table 2.** For each COVID-19 distribution the preferred PDF with the largest Bayesian support is listed, along with the estimated mean, variance and other parameters of the PDF. Ninety-five per cent credible intervals are given in brackets for mean and variance. The parameters *p*_1_, *p*_2_ and *p*_3_ for the preferred PDFs gamma and generalized lognormal (GLN) are given in the form gamma(*x*|*p*_1_, *p*_2_) = gamma(*α*, *β*) and GLN(*x*|*p*_1_, *p*_2_, *p*_3_) = GLN(*μ*, *σ*, *s*), with the formulae of the PDFs given in appendix B, table 4. The credible intervals for parameters *p*_1_, *p*_2_ and *p*_3_ are less than 0.1 wide, so only the point estimates are shown.

distribution	preferred PDF	mean (days)	variance (days^2^)	*p*_1_	*p*_2_	*p*_3_
onset-to-death	gamma	15.2 (15.1, 15.3)	105.3 (103.7, 106.9)	2.2	0.1	—
hospital-admission-to-death	gamma	10.0 (9.9, 10.0)	84.8 (83.2, 86.4)	1.2	0.1	—
ICU-stay	gamma	9.0 (8.9, 9.1)	64.9 (63.1, 66.8)	1.2	0.1	—
onset-to-hospital-admission	gen. lognormal	7.8 (7.7, 7.8)	35.7 (35.0, 36.5)	1.8	0.6	1.8
onset-to-hospital-discharge	gen. lognormal	17.6 (17.6, 17.7)	248.7 (233.7, 265.6)	2.7	0.3	1.2
onset-to-ICU-admission	gen. lognormal	8.5 (8.4, 8.5)	48.0 (46.1, 50.0)	1.9	0.6	1.8
onset-to-diagnosis (PCR)	gen. lognormal	12.5 (12.5, 12.6)	252.3 (236.4, 269.6)	2.3	0.3	1.2
onset-to-diagnosis (non-PCR)	gen. lognormal	14.5 (14.3, 14.7)	†	2.3	0.3	1.0

^†^The variance diverges for the onset-to-diagnosis (non-PCR) PDF.

Additionally, in figure 2, in each instance the cumulative probability distribution is given for the best model fit, revealing that out of patients for whom COVID-19 is terminal, almost 70% die within 20 days of symptom onset. Out of patients who die in the hospital, almost 60% die within the first 10 days since admission.

The estimated mean number of days for each distribution for Brazil is compared in table 3 with values found in the literature for China, USA and France. The majority of the data obtained through searching the literature pertained to the early stages of the epidemic in China, and no data were found for low- and middle-income countries. The mean onset-to-death time of 15.2 (95% CrI 15.1–15.3) days, from a best-fitting gamma PDF, is shorter than the 17.8 (95% CrI 16.9–19.2) days estimate from Verity *et al.* [6] and 20.2 (95% CrI 15.1–29.5) days estimate (14.5 days without truncation) from Linton *et al.* [12] In both cases, estimates were based on a small sample size from the beginning of the epidemic in China. The mean number of days for hospital-admission-to-death of 10.8 (95% CrI 10.7–10.9) for Brazil matches closely the 10 days estimated by Salje *et al.* [32]

**Table 3.** Epidemiological distributions for COVID-19 for Brazil, China, France and USA. PDF means for Brazil have been obtained using Markov chain Monte Carlo (MCMC) sampling, using the PDF with the maximum Bayesian support for each data distribution (see appendix B, table 6). For China, France and USA, the sources have been obtained from the literature. All values are given in days, and 95% CrI are given in brackets unless stated otherwise.

distribution	Brazil	China	France	USA
onset-to-death	15.2 (15.1, 15.3)	17.8 (16.9, 19.2) [6]		13.59^b^ (7.85) [33]
	16.0* (15.9, 16.1)	18.8* (15.7, 49.7) [6]		
		14.5 (12.5, 17.0) [12]		
		20.2* (15.1, 29.5) [12]		
hospital-admission-to-death	10.0 (9.9, 10.0)	5.0^a^ (3.0, 9.3) [34]	10.0 [35]	
	10.8* (10.7, 10.9)	8.9 (7.3−10.4) [12]		
		13.0* (8.7−20.9) [12]		
ICU-stay	9.0 (8.9, 9.1)	8.0^a^ (4.0, 12.0) [36]	17.6 (17.0, 18.2) [35]	
	10.1* (9.9, 10.2)			
onset-to-hospital-admission	7.8 (7.7, 7.8)	10.0^a^ (7.0–12.0) [34]		
onset-to-hospital-discharge	17.6 (17.6, 17.7)	22.0^a^ (18.0, 25.0) [36]		
onset-to-ICU-admission	8.5 (8.4, 8.5)	9.5^a^ (7.0, 12.5) [37]		
onset-to-diagnosis	12.5^†^(12.5, 12.6)	5.5 (5.4, 5.7) [32]		
	14.5^‡^(14.3, 14.7)	5.5 (5.4, 5.7) [32]		

*Adjusted for censoring, ^†^PCR confirmed, ^‡^non-PCR confirmed, ^a^Median (interquartile range), ^b^Mean (standard deviation).

### 3.2. Subnational Brazilian epidemiological distributions

**Figure 8.**
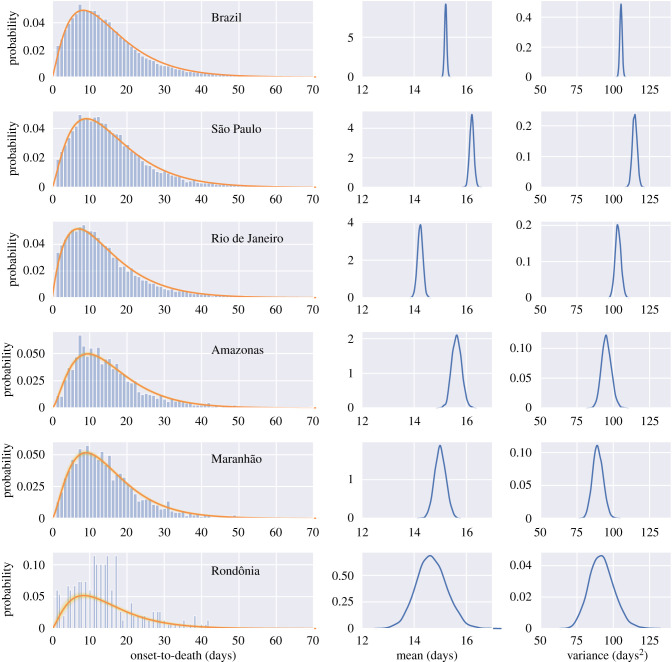
Gamma PDF gamma(*α*, *β*) fitted to the onset-to-death data for Brazil and five states of Brazil. The PDFs were fitted with HMC partially pooling each state with the whole country. The red lines represent the model using the mean parameter estimates. Individual PDFs selected during MCMC sampling are shown in yellow. Posterior mean and variance distributions for each region are given in the middle and right-hand side columns.

The onset-to-death distribution, and other time-delay distributions such as onset-to-diagnosis, length of ICU stay, onset-to-hospital-admission, onset-to-hospital-discharge, onset-to-ICU-admission and hospital-admission-to-death, have been fitted in a joint model across the 26 states and one federal district of Brazil using partial pooling. The mean number of days, plotted in figure 3, shows substantial subnational variability—for example, the mean onset-to-hospital-admission for Amazonas state was estimated to be 9.9 days (95% CrI 9.7–10.1), whereas for Mato Grosso do Sul the estimate was 6.7 (95% CrI 6.4–7.1) days and Rio de Janeiro - 7.2 days (95% CrI 7.1–7.3). Amazonas state had the longest average time from onset-to-hospital- and ICU-admission. The state with the shortest average onset-to-death time was Roraima. Santa Catarina state on the other hand had a longest average onset-to-death and hospital-admission-to-death time, as well as longest average ICU-stay. For a visualization of the uncertainty in our mean estimates for each state, see the posterior density plots in appendix B, figures 6 and 7. Additional national and state-level results for the onset-to-death gamma PDF, including the posterior plots for mean and variance, are shown in appendix B, figure 8.

We also observe discrepancies between the five geographical regions of Brazil, for example states belonging to the southern part of the country (Paraná, Rio Grande do Sul and Santa Catarina) had a longer average ICU-stay and hospital-admission-to-death time when compared with the states in the North region. Full results, including detailed estimates of mean, variance, and estimates for each of the distributions’ parameters for Brazil and Brazilian states can be accessed at https://github.com/mrc-ide/Brazil_COVID19_distributions/blob/master/results/results_full_table.csv.

### 3.3. Sensitivity analyses

**Figure 9.**
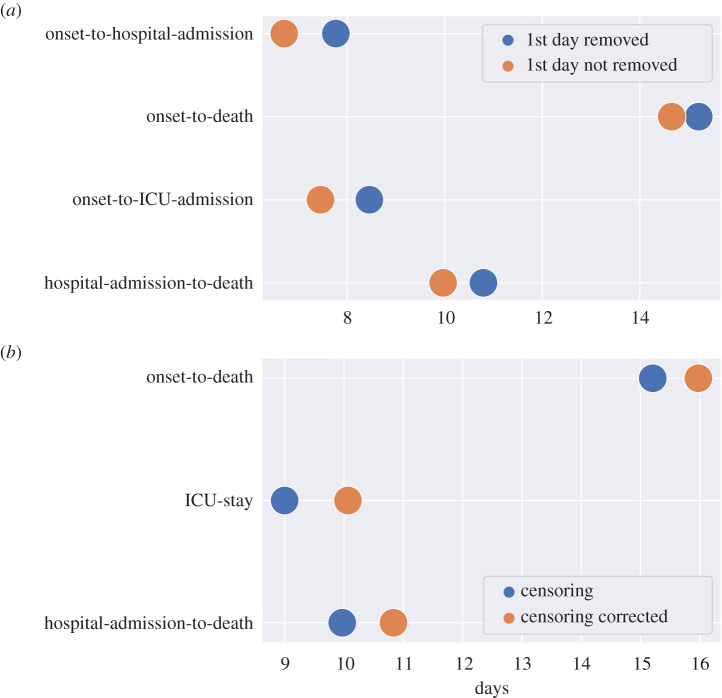
Estimated mean per distribution in different scenarios: excluding first day data points (*a*) and censoring correcting (*b*). The credible intervals were not shown as due to the large amount of data available they were negligible.

In order to remove the potential bias towards shorter outcomes from left- and right-censoring, we tested the scenario in which the data to fit the models were truncated. For example, based on a 95% quantile of 35 days for the hospital-admission-to-death distribution, entries with the starting date (hospital admission) after 2 June 2020 and those with an end-date (death) before 1 April 2020 were truncated, and the models were refitted. With censored parts of the data removed, the mean time from start to outcome increased for every distribution, e.g. for hospital-admission-to-death it increased from 10.0 days (95% CrI 9.9–10.0) to 10.8 (95 % CrI 10.7–10.9), and for onset-to-death it changed from 15.2 days (95% CrI 15.1–15.3) to 16.0 (95% CrI 15.9–16.1). The effect of truncation on censored data is given in appendix B, figure 9.

To test the impact of keeping or removing entries identified as potentially resulting from erroneous data transcription (see the methods §2), we fitted the PDFs to some of the distributions on a national level with and without those entries. For onset-to-hospital-admission, onset-to-ICU and onset-to-death we find that generalized gamma PDF was preferred when the first day of the distribution was included, and gamma (for onset-to-death) and generalized lognormal PDFs if the first day was removed. For hospital-admission-to-death, a gamma distribution fitted most accurately when the first day was included, and Weibull when it was excluded. Removing the first day results in the mean values shifting to the right by approximately 1 day for both onset-to-hospital- and ICU-admission, and by 0.5 days for hospital-admission-to-death (see appendix B, figure 9).

Sensitivity analysis regarding the model selection approach is detailed in appendix A.

## 4. Discussion

We fitted multiple probability density functions to a number of epidemiological datasets, such as onset-to-death or onset-to-diagnosis, from the Brazilian SIVEP Gripe database [13], using Bayesian hierarchical models. Our findings provide the first reliable estimates of the various epidemiological distributions for the COVID-19 epidemic in Brazil and highlight a need to consider a wider set of specific parametric distributions. Instead of relying on the ubiquitous gamma or lognormal distributions, we show that often these PDFs do not best capture the behaviour of the data. For instance, the generalized lognormal is preferable for several of the epidemiological distributions in table 2. These results can specifically inform modelling of the epidemic in Brazil [38], and other low- and middle-income countries [39], but we expect they are also highly relevant to the epidemics unfolding in other countries.

Across Brazil, the epidemic has strong geographical heterogeneity, with some states such as Amazonas and Maranhão reported to be at advanced stages [40,41]. To describe the observed differences at subnational level accurately, using a mathematical model, it is essential to account for variation in model parameters by state. By making use of the state-level custom-fitted onset-to-death distributions reported here, we have estimated the number of active infections on 23 June 2020 across 10 states spanning the five regions of Brazil using a Bayesian hierarchical renewal-type model [7,38,42]. The relative change in the number of active infections from modelling the cases using heterogeneous state-specific onset-to-death distributions, compared to using a single common Brazil one, is shown in figure 4 to be substantial. Relative changes are observed of up to 18% more active infections, suggesting common assumptions of onset-to-death spatial homogeneity are unreliable and closer attention needs to be paid when fitting models of SARS-CoV-2 transmission dynamics in large countries.

Notably, large subnational variability was observed for all fitted distributions, with the mean onset-to-death ranging between 11.2 days in Roraima to 17.8 in Santa Catarina. Hospital-admission-to-death time showed substantial variation between the regions of Brazil, ranging between 8.1 and 11.3 in the North, and between 9.6 and 12.8 in the South. A plausible hypothesis is that the observed differences in outcome timings could be explained by greater difficulty accessing hospitals in the North, or limited access to equipment such as ventilators.

In order to explain the origin of the geographical variation of average distribution times across states, shown in figure 3, we present a basic exploratory analysis based on relevant high-level features. We examined the correlation between socio-economic factors, such as education, poverty, income, wealth, deprivation and segregation, using a number of socio-economic state-level indicators obtained from Barrozo *et al*. [43] and additional datasets containing the mean age per state and percentage of people living in the urban areas (urbanicity) [44]. The Pearson correlation coefficients, shown in appendix B, table 8, suggest that poverty, income, segregation and deprivation elements were most strongly correlated with the analysed onset-time datasets. In particular, poverty was strongly negatively correlated with hospital-admission-to-death (−0.68), whereas income and segregation had a high positive correlation coefficient for the same distribution (+0.60, +0.62, respectively). The strongest correlation was observed for hospital-admission-to-death and deprivation indicator, which measures the access to sanitation, electricity and other material and non-material goods [43]. Interestingly, the indicators measuring economic situation were more correlated with average hospitalization times than mean age per state, which suggests that although the low- and middle-income countries typically have younger populations, their healthcare systems are more likely to struggle in response to the COVID-19 epidemic. Socio-economic factors have been also shown to correlate with the accessibility of the COVID-19 diagnosis in the Metropolitan Region of São Paulo, which emphasizes the impact of the spatial heterogeneity of the socio-economic status on the various aspects of the epidemic, from capturing the active cases to providing treatment for the patients [14]. More detailed analysis is necessary to fully appreciate the impact of the economic components on the COVID-19 epidemic response.

Spatial heterogeneity is not the only source of variability in the hospitalization times. Although in this study, we did not stratify the population according to age or other demographic features, other recent studies have used the SIVEP-Gripe database to characterize the COVID-19 epidemic in Brazil. Namely, they looked at the regional and ethnic distribution of the hospitalized patients [15,16], age-sex structure and clinical characteristics such as co-morbidities and symptoms [14,15]. Souza *et al.* [14] show that 65.5% of cases are patients over 50 years old. Moreover, they also find that 84% of the patients reported having at least one underlying condition. It is clear, that both age and co-morbidities are highly correlated with the adverse outcomes such as hospitalization or death, and to calibrate the epidemiological models of COVID-19 the time-onset distributions presented in this study could be refined further.

In the work presented, we acknowledge several limitations. The database from which distributions have been extracted, though extensive, contains transcription errors, and the degree to which these bias our estimates is largely unknown. Secondly, the PDFs fitted are based on observational hospital data, and therefore should be cautiously interpreted for other settings. Thirdly, though we have fitted PDFs at subnational as well as national level, this partition is largely arbitrary and further work is required to understand the likely substantial effect of age, sex, ethnic variation, co-morbidities and other factors.

## 5. Conclusion

We provide the first estimates of common epidemiological distributions for the COVID-19 epidemic in Brazil, based on the SIVEP-Gripe hospitalization data [13]. Extensive heterogeneity in the distributions between different states is reported. The differences are identified by comparing parametric forms, that have been fitted for each epidemiological distribution, and give a more informed and reliable basis for comparison than the empirical distributions. Quantifying the time-delay for COVID-19 onset and hospitalization data provides useful input parameters for many COVID-19 epidemiological models, especially those modelling the healthcare response in low- and middle-income countries.

## Data accessibility

Python, R and Stan code used to analyse the data and fit the distribution is available at https://github.com/mrc-ide/Brazil_COVID19_distributions, along with estimated parameters for each state and PDFs considered at https://github.com/mrc-ide/Brazil_COVID19_distributions/blob/master/results/results_full_table.csv. The SIVEP-Gripe database [13] is available to download from the Brazil Ministry of Health website https://opendatasus.saude.gov.br/dataset/bd-srag-2020.

## Appendix A. Model selection

To characterize which model (gamma, lognormal etc.) best fits the data, the Bayesian model evidence *z* = *z*(*y*|*M*_*i*_) is evaluated. Here and throughout this section, *y* denotes the data and *M*_*i*_ denotes the *i*^th^ model from the analysed model set. As determining the model evidence requires calculating an integral over the model parameters (*θ*) which is generally intractable, we approximate it with *z*_0_ = *z*_0_(*y*|*M*_*i*_), which is based on a second-order Laplace approximation [45], *q*_0_ = *q*_0_(*θ*|*M*_*i*_, *y*), to the true un-normalized posterior density *q* = *q*(*θ*|*M*_*i*_, *y*). The second-order approximated density is estimated as

A1 $$q_{0}=q(\;\hat{\theta }\;)\;\exp\left(-\frac{1}{2}\;(\theta -\hat{\theta }\;)\;{\hat{\Sigma }}^{-1}\;{\left(\theta -\hat{\theta }\;\right)}^{T}\right).\;(A\;1)$$

Here, $q(\;\hat{\theta }\;)$ denotes the value of the un-normalized posterior evaluated using the mean estimates of the model’s parameters $\hat{\;\theta \;}$, and $\hat{\Sigma }$ the covariance matrix built from MCMC samples of the posterior distribution. From this expression, a second-order approximation to the model evidence, *z*_0_, is given by $z_{0}=q(\;\hat{\theta }\;)\sqrt{\text{det}(2\pi {\hat{\Sigma }}^{-1})}$, where det( · ) denotes the determinant of the matrix.

For each model pair, BFs were computed from the marginal likelihoods. Considering two models *M*_*i*_ and *M*_*j*_, the BF is

A2 $$B_{ij}=\frac{z(y|M_{i})}{z(y|M_{j})},\;(A\;2)$$

where *z*(*y*|*M*_*i*_) is the evidence of model *M*_*i*_ given *y*. If *B*_*ij*_ > 1, the evidence is in favour of model *M*_*i*_. Here, for readability, we will report the BFs as 2 log(*B*_*ij*_) following Kass and Raftery notation [46].

The sensitivity of our model evidence is tested with respect to the choice of hyperprior distribution, and secondly with respect to the use of the approximate second-order density *q*_0_. In the latter instance, this is done by performing thermodynamic integration [27,30,31] between *q*_0_ and the true density *q* in order to obtain an asymptotically exact estimate of the marginal model evidence,

A3 $$z=z_{0}\text{exp}\left(\int_{0}^{1}E_{\theta ∼q(\theta ;\;\lambda )}[\text{log}\;q_{}-\text{log}\;q_{0}]\text{d}\lambda \right).\;(A\;3)$$

The right-hand term corrects the *z*_0_ approximation to the exact Bayesian evidence by a path integral evaluated with respect to a sampling distribution that interpolates between the two densities as $q(\theta ;\;\lambda )=q^{\left(1-\lambda \right)}q_{0}^{\lambda }$ in terms of the auxiliary coordinate *λ*.

## Appendix B

See additional tables (4–9) and figures (5–9)

**Table 4.** Probability density functions with analytical formulae for mean and variance. *y* denotes the data, *Γ*( · ) is a gamma function. GG, generalized gamma [20]; GLN, generalized log-normal [19].

PDF	mean	variance
$\text{gamma}(y|\alpha ,\beta )=\frac{\beta^{\alpha }}{\Gamma (\alpha )}y^{\alpha -1}\exp(-\beta y)$	$\frac{\alpha }{\beta }$	$\frac{\alpha }{\beta^{2}}$
$\text{Weibull}(y|\alpha ,\sigma )=\frac{\alpha }{\sigma }{\left(\frac{y}{\sigma }\right)}^{\alpha -1}\exp\left(-{\left(\frac{y}{\sigma }\right)}^{\alpha }\right)$	$\sigma \Gamma \left(1+\frac{1}{\alpha }\right)$	$\sigma^{2}\left(\Gamma \left(1+\frac{2}{\alpha }\right)-\Gamma^{2}\left(1+\frac{1}{\alpha }\right)\right)$
$\text{lognormal}(y|\mu ,\sigma )=\frac{1}{\sqrt{2\pi }\sigma }\frac{1}{y}\exp\left(-\frac{1}{2}{\left(\frac{\log y-\mu }{\sigma }\right)}^{2}\right)$	$\text{exp}\left(\mu +\frac{\sigma^{2}}{2}\right)$	(exp(*σ*^2^) − 1)exp (2*μ* + *σ*^2^)
$\text{GG}(y|a,d,p)=\frac{1}{\Gamma (d/\;p)}{\left(\;p/a\right)}^{d}x^{d-1}\exp\left(-{\left(\frac{y}{a}\right)}^{p}\right)$	$a\frac{\Gamma ((d+1)/p)}{\Gamma (d/p)}$	$a^{2}\left[\frac{\Gamma ((d+2)/p)}{\Gamma (d/p)}-{\left(\frac{\Gamma ((d+2)/p)}{\Gamma (d/p)}\right)}^{2}\right]$
$\text{GLN}(y|\mu ,\sigma ,s)=\frac{1}{y}\frac{s}{2^{(s+1)/s}\sigma \Gamma (1/s)}\exp\left(-\frac{1}{2}{\left|\frac{\log y-\mu }{\sigma }\right|}^{s}\right)$	$\text{exp}(\mu )\left[1+\frac{S}{2\Gamma (1/s)\;}\right],$ $S=\sum_{\;j=1}^{\infty }\sigma^{\;j}(1+{\left(-1\right)}^{\;j})2^{\;j/s}\frac{\Gamma ((\;j+1)/2)}{\Gamma (j+1)}$	$\text{exp}(2\mu )\left[1+\frac{S}{2\Gamma (1/s)}\right]-{\left[\text{Mean}\right]}^{2}$, $S=\sum_{\;j=1}^{\infty }2\sigma^{\;j}(1+{\left(-1\right)}^{\;j})2^{\;j/s}\frac{\Gamma ((\;j+1)/2)}{\Gamma (j+1)}$

**Table 5.** Number of datapoints per state for each of the datasets analysed in the study. Acre (AC), Alagoas (AL), Amazonas (AM), Amapá (AP), Bahia (BA), Ceará (CE), Distrito Federal (DF), Espírito Santo (ES), Goiás (GO), Maranhão (MA), Minas Gerais (MG), Mato Grosso do Sul (MS), Mato Grosso (MT), Pará (PA), Paraíba (PB), Pernambuco (PE), Piauí (PI), Paraná (PR), Rio de Janeiro (RJ), Rio Grande do Norte (RN), Rondônia (RO), Roraima (RR), Rio Grande do Sul (RS), Santa Catarina (SC), Sergipe (SE), São Paulo (SP) and Tocantins (TO).

	onset-death	admission-death	ICU-stay	onset-hospital admission	onset-hospital discharge	onset-ICU admission	onset-diagnosis (PCR)	onset-diagnosis (non-PCR)
AC	239	115	2	225	4	9	345	1
AL	1040	894	680	1600	629	859	1344	416
AM	2736	2403	1010	5971	2573	1323	4502	1604
AP	181	175	68	299	136	80	183	153
BA	2241	2013	982	4563	1338	2300	5266	352
CE	5801	4905	1534	9685	4536	2768	8286	1749
DF	662	655	499	2687	1415	1198	2864	311
ES	1292	1023	589	1409	507	778	1774	321
GO	698	637	375	1813	783	819	2018	122
MA	1950	1097	197	1485	247	341	1562	821
MG	1223	1176	603	4782	2210	1521	4910	604
MS	131	124	46	723	417	171	764	126
MT	286	248	83	1347	2191	384	4695	2175
PA	4727	3934	1270	8226	3034	1993	6921	1351
PB	1136	1037	349	1992	508	740	1584	644
PE	4408	3284	311	6574	1888	1566	9745	190
PI	515	497	139	2161	341	490	2314	240
PR	793	773	898	3174	1952	1168	3490	124
RJ	9750	9068	1490	18 019	7438	7165	21 159	1446
RN	876	821	337	1878	664	693	1517	544
RO	254	238	180	554	180	284	488	293
RR	270	265	53	98	51	56	200	92
RS	790	770	971	3565	2328	1277	4144	477
SC	408	389	291	1600	777	599	1634	343
SE	303	295	193	938	181	306	1116	117
SP	16 348	15 808	8515	55 735	32 937	17 642	63 184	4769
TO	213	177	44	515	213	87	549	53

**Table 6.** Bayes factors (BFs) for the analysed distributions and models. For each distribution (rows), the values represent BF for the best-fitting model against other models. Value of 0 indicates the model that fits the data the best. Value > 10 indicates a very strong evidence against given model compared to the best one. GLN, generalized log-normal; GG, generalized gamma; NA, not analysed. The BF values are reported here as 2 log(*B*_*ij*_) following Kass & Raftery notation [46].

	gamma	Weibull	lognormal	GLN	GG
onset-to-death	0	2156	2208	198	301
admission-death	0	195	4349	3096	188
ICU-stay	0	231	588	607	352
onset-to-hospital-admission	4000	17 073	494	0	NA
onset-to-hospital-discharge	2819	8346	6079	0	3087
onset-to-ICU-admission	798	4359	142	0	1244
onset-to-diagnosis (PCR)	1111	10 400	13 882	0	1257
onset-to-diagnosis (non-PCR)	578	793	4340	0	461

**Table 7.** State-level onset-to-death estimates for gamma PDF: mean, variance, parameters values, with 95% credible intervals. The parameters *p*_1_ and *p*_2_ are given in the form gamma(*x*|*p*_1_, *p*_2_) = gamma(*α*, *β*). The full PDFs for other distributions are available at https://github.com/mrc-ide/Brazil_COVID19_distributions/blob/master/results/results_full_table.csv.

state	mean (days)	variance (days^2^)	*p*_1_	*p*_2_
AC	17.4 (16.1, 18.8)	119.4 (98.8, 143.6)	2.6 (2.2, 2.9)	0.1 (0.1, 0.2)
AL	14.0 (13.4, 14.5)	82.5 (74.3, 91.9)	2.4 (2.2, 2.5)	0.2 (0.2, 0.2)
AM	15.6 (15.3, 16.0)	95.3 (89.1, 102.1)	2.6 (2.4, 2.7)	0.2 (0.2, 0.2)
AP	14.5 (13.2, 16.0)	99.1 (79.8, 122.7)	2.1 (1.9, 2.4)	0.1 (0.1, 0.2)
BA	15.1 (14.7, 15.6)	116.6 (107.9, 126.1)	2.0 (1.9, 2.1)	0.1 (0.1, 0.1)
CE	16.1 (15.8, 16.4)	116.4 (111.1, 122.0)	2.2 (2.2, 2.3)	0.1 (0.1, 0.1)
DF	16.4 (15.6, 17.2)	105.0 (92.7, 119.0)	2.6 (2.3, 2.8)	0.2 (0.1, 0.2)
ES	17.0 (16.4, 17.5)	107.8 (98.2, 118.1)	2.7 (2.5, 2.9)	0.2 (0.1, 0.2)
GO	14.5 (13.8, 15.2)	87.9 (77.9, 99.1)	2.4 (2.2, 2.6)	0.2 (0.2, 0.2)
MA	15.0 (14.6, 15.4)	89.4 (82.7, 96.5)	2.5 (2.4, 2.7)	0.2 (0.2, 0.2)
MG	15.1 (14.6, 15.7)	95.1 (86.3, 104.7)	2.4 (2.2, 2.6)	0.2 (0.1, 0.2)
MS	14.8 (13.3, 16.4)	93.9 (74.8, 116.8)	2.4 (2.0, 2.7)	0.2 (0.1, 0.2)
MT	14.1 (13.1, 15.1)	80.6 (67.2, 96.4)	2.5 (2.2, 2.8)	0.2 (0.2, 0.2)
PA	14.7 (14.5, 15.0)	90.2 (85.7, 94.9)	2.4 (2.3, 2.5)	0.2 (0.2, 0.2)
PB	14.0 (13.4, 14.5)	78.7 (71.2, 87.3)	2.5 (2.3, 2.7)	0.2 (0.2, 0.2)
PE	13.0 (12.7, 13.2)	89.7 (84.6, 95.1)	1.9 (1.8, 1.9)	0.1 (0.1, 0.2)
PI	16.5 (15.6, 17.4)	114.8 (99.4, 131.7)	2.4 (2.1, 2.6)	0.1 (0.1, 0.2)
PR	15.7 (15.1, 16.4)	91.9 (81.8, 102.7)	2.7 (2.5, 2.9)	0.2 (0.2, 0.2)
RJ	14.2 (14.0, 14.4)	103.3 (99.5, 107.3)	2.0 (1.9, 2.0)	0.1 (0.1, 0.1)
RN	15.2 (14.6, 15.9)	91.9 (81.8, 103.0)	2.5 (2.3, 2.7)	0.2 (0.2, 0.2)
RO	14.7 (13.6, 15.8)	92.1 (76.4, 110.0)	2.3 (2.1, 2.6)	0.2 (0.1, 0.2)
RR	11.2 (10.2, 12.1)	68.1 (55.9, 83.0)	1.8 (1.6, 2.1)	0.2 (0.1, 0.2)
RS	15.4 (14.7, 16.2)	116.0 (103.0, 130.8)	2.1 (1.9, 2.2)	0.1 (0.1, 0.1)
SC	17.8 (16.7, 19.0)	146.8 (125.1, 173.5)	2.2 (1.9, 2.4)	0.1 (0.1, 0.1)
SE	13.4 (12.2, 14.5)	112.5 (91.4, 138.6)	1.6 (1.4, 1.8)	0.1 (0.1, 0.1)
SP	16.2 (16.0, 16.4)	114.8 (111.6, 118.0)	2.3 (2.2, 2.3)	0.1 (0.1, 0.1)
TO	14.8 (13.5, 16.2)	97.3 (79.1, 119.7)	2.3 (2.0, 2.6)	0.2 (0.1, 0.2)
Brazil	15.2 (15.1, 15.3)	105.3 (103.7, 106.9)	2.2 (2.2, 2.2)	0.1 (0.1, 0.1)

**Table 8.** Pearson correlation coefficients for mean distribution times and socio-economic indicators. Sample size was equal to 27 (number of states).

	ICU-stay	onset-death	admission-death	onset-discharge	onset-hospital admission	onset-ICU admission	onset-diagnosis (PCR)
education	−0.32	−0.25	−0.62	0.41	0.48	0.39	0.34
poverty	−0.31	−0.31	−0.68	0.52	0.69	0.54	0.49
deprivation	0.38	0.35	0.71	−0.49	−0.59	−0.49	−0.41
wealth	−0.08	0.26	0.37	−0.24	−0.07	−0.21	-0.17
income	0.21	0.28	0.60	−0.35	−0.40	−0.33	−0.35
segregation	0.40	0.35	0.62	−0.43	−0.57	−0.47	−0.30
mean age	0.13	0.25	0.43	−0.45	−0.57	−0.68	−0.25
urbanicity	0.12	0.11	0.43	−0.34	−0.52	−0.40	−0.19

**Table 9.** Pearson correlation coefficients for mean distribution times. Sample size was equal to 27 (number of states).

	onset-death	admission-death	onset-discharge	onset-hospital admission	onset-ICU admission	onset-diagnosis (PCR)
onset-death	1	0.69	−0.35	0.06	0.24	0.15
admission-death	0.69	1	−0.52	−0.48	−0.20	−0.36
onset-discharge	−0.35	−0.52	1	0.39	0.43	0.40
onset-to-hospital-admission	0.06	−0.48	0.39	1	0.72	0.53
onset-to-ICU-admission	0.24	−0.20	0.43	0.72	1	0.50
onset-to-diagnosis (PCR)	0.15	−0.36	0.40	0.53	0.50	1
